# The Effect of Modulation Enhancement Scheme on Speech Recognition in Spatial Noise Among Young Adults with Normal Hearing

**DOI:** 10.3390/audiolres16010026

**Published:** 2026-02-14

**Authors:** Vibha Kanagokar, Yashu MA, Jayashree S. Bhat, Arivudai Nambi Pitchaimuthu

**Affiliations:** 1 Department of Audiology and Speech Language Pathology, Kasturba Medical College Mangalore, Manipal Academy of Higher Education, Manipal, India; vibha.kanagokar13@gmail.com; 2 Department of Speech and Hearing, SDM College of Medical Sciences and Hospital, Dharwad 580009, Karnataka, India; yashumasuma@gmail.com; 3 Kasturba Medical College Hospital, Ambedkar Circle, Mangalore, Karnataka, India; bhatsjayashree@gmail.com; 4 Centre for Hearing Sciences, Department of Audiology, All India Institute of Speech and Hearing, Mysore 570006, Karnataka, India

**Keywords:** temporal envelope, envelope expansion, envelope enhancement, spatial release from masking, interaural time difference, interaural coherence, speech perception in noise, modulation depth, spatial hearing, normal-hearing adults

## Abstract

**Background/Objectives**: Speech understanding in noise relies on both temporal fine structure (TFS) and temporal envelope (ENV) cues. While TFS primarily conveys interaural time differences (ITDs) at low frequencies, ENV cues can also support ITD processing, especially when TFS is unavailable or degraded. Expanding the ENV by increasing modulation depth has been proposed to improve speech perception, but its effects on spatial release from masking (SRM) and binaural temporal processing in normal-hearing listeners remain unclear. The goal of this study was to evaluate the effect of ENV enhancement on SRM in young adults with normal hearing and its influence on ITD sensitivity and interaural coherence (IC). **Method**: Thirty normal-hearing native Kannada speakers (19–34 years) participated. Speech stimuli consisted of Kannada sentences embedded in four-talker babble at −5, 0, and +5 dB signal to noise ratio (SNR). Target and masker were spatialized using head-related transfer functions at 0°, 15°, and 37.5° azimuths. Stimuli were presented with and without ENV enhancement (compression–expansion algorithm). Speech recognition scores were analyzed using generalized linear mixed models, and SRM was calculated as performance differences between co-located and spatially separated conditions. Cross-correlation analyses were performed to estimate ITDs and IC across SNRs. **Result**: ENV enhancement yielded significantly higher SRM values across all SNRs and spatial separations. Benefits were greatest at lower SNRs and wider target–masker separations. Cross-correlation analysis showed enhanced IC and more reliable ITD estimates under the expanded condition, particularly at moderate SNRs. **Conclusions**: Temporal ENV enhancement strengthens spatial unmasking and binaural timing cues in normal-hearing adults, especially under adverse listening conditions. These findings highlight its potential application in auditory rehabilitation and hearing technologies where ENV cues are critical.

## 1. Introduction

Understanding speech in noisy environments is a complex auditory task that challenges both individuals with normal hearing and those with hearing impairments. In everyday situations such as social gatherings, classrooms, or public spaces, multiple sound sources often compete for attention. Under these conditions, listeners rely on spatial hearing to focus on a target speaker while suppressing competing background sounds, a phenomenon often described as the “cocktail party effect” [1,2]. A key mechanism supporting this selective attention is spatial release from masking (SRM), which refers to the improvement in speech recognition when the target and masker originate from different spatial locations. SRM is mediated by binaural cues, particularly interaural time differences (ITDs) and interaural level differences (ILDs), which enable the auditory system to localize sounds and selectively attend to a desired source [3,4].

Successful speech perception in noise depends not only on access to spatial cues but also on the integrity of the speech signal itself, especially its temporal structure. The temporal envelope (ENV), which represents the slower amplitude variations in a speech waveform, encodes critical information about syllabic rhythm and prosody. These cues are particularly important when temporal fine structure (TFS) is inaccessible or poorly represented, as in cochlear implant (CI) users or individuals with cochlear hearing loss or auditory neuropathy [5,6,7]. Beyond speech intelligibility, ENV cues also contribute to spatial hearing by supporting ITD detection and source segregation in the absence of TFS cues [8]. At higher frequencies, where TFS is degraded due to neural phase locking limits, ENV based ITDs provide an alternative spatial cue that, in well-structured signals, can yield sensitivity comparable to low frequency TFS [8,9]. Spatial accuracy and the benefits of SRM are greatest when the ENV has pronounced modulation patterns, particularly with sharp onsets, whereas mismatches or inconsistencies in ENV timing across ears diminish spatial perception [10,11]. Rapid onset segments of the ENV are especially important, as listeners often use these brief acoustic “glimpses” to recover spatial details in fluctuating or reverberant listening situations [12]. Neuroimaging studies further demonstrate that ENV based ITDs contribute to spatial coding in the auditory cortex, particularly in the high-frequency range [13].

Although ENV cues are generally sufficient for effective speech understanding in quiet, their robustness diminishes in noisy or reverberant environments. Background noise and reflections can reduce modulation depth, fill in the amplitude dips, and distort the ENVs shape, thereby impairing neural encoding and binaural processing. Such degradation compromises the detection of ITDs and the maintenance of interaural coherence (IC), both essential for spatial hearing [14,15,16]. Differences in ENV across ears, due to noise or asymmetric processing, further reduces interaural similarity thereby limiting localization accuracy [16]. Additionally, noise can weaken neural phase-locking to the ENV, especially at low modulation depths or in the presence of interaural disparities [15,17]. These challenges are particularly pronounced for hearing aid (HA) and CI users, where independent compression in each device may further disrupt interaural ENV coherence, thereby reducing spatial perception.

To mitigate the negative effects of noise on ENV cues, various signal processing strategies have been explored to enhance speech modulations. One such approach is ENV enhancement, which increases modulation depth to make important speech cues more prominent. This technique has shown promise in telecommunications and audiology, including studies demonstrating improved speech perception in noise for individuals with auditory neuropathy and sensorineural hearing loss [7,18]. Other research has indicated that ENV enhancement can improve consonant recognition, decrease response times, and lower speech identification thresholds under difficult listening conditions [19,20]. A non-linear ENV enhancement method across frequency bands has been shown to provide modest improvement at 0 dB signal to noise ratio (SNR) [21]. Deep Band Modulation (DBM), which enhances modulation depth and temporal structure, significantly improves speech perception in individuals with temporal processing deficits [22]. Similarly, optimal nonlinear amplitude mapping in CI users can also improve vowel and consonant recognition, supporting the efficacy of tailored ENV enhancement techniques [23].

Despite growing evidence for the benefits of ENV enhancement, its specific impact on SRM and spatial hearing has not been fully investigated. Prior studies have emphasized the role of ENV cues in vocoded or simulated CI conditions, where increasing the number of channels conveying independent ENV information led to better SRM [24]. Additionally, some findings suggest that even low-frequency components of the ENV may be sufficient to support spatial unmasking in certain conditions [25,26]. However, no study to date has directly assessed whether enhancing ENV modulations in natural speech improves SRM and binaural temporal processing such as ITD sensitivity IC in individuals with normal hearing.

This area of investigation is not only important for understanding normal auditory function but also has potential applications in clinical populations. Older adults and individuals with hearing loss often experience reduced temporal resolution and neural synchrony, which can impair their ability to benefit from spatial cues and modulated speech signals [27,28]. Enhancing ENV modulations may help compensate for these deficits, offering a pathway toward improved spatial hearing in real-world environments. This technique may also be relevant for modern hearing devices, many of which do not transmit TFS information and instead rely on ENV-based processing to deliver speech signals.

The current study was conducted to examine whether enhancing the ENV of speech improves SRM and binaural temporal processing in normal-hearing young adults. This line of investigation builds on our previous work in which we showed that variations in interaural modulation depth significantly influence ITD thresholds for speech, thereby highlighting the role of ENV characteristics in shaping binaural timing sensitivity [29]. If proven effective, this enhancement strategy could have valuable applications in hearing technology and auditory rehabilitation, particularly for populations that depend on ENV cues due to impaired temporal coding.

## 2. Materials and Methods

### 2.1. Participants

30 young adults in the age range 19–34 years (mean age = 25 ± 4.2 years) participated in the present study. All were native Kannada speakers and had their pure tone hearing thresholds ≤ 20 dB HL from 0.25 to 8 kHz. The demographic details of the participants are provided in Supplementary Table S1. The study was approved by the Institutional Ethics Committee of Kasturba Medical College, Mangalore, India (Reference No: IEC KMC MLR 12-18/503). Informed written consent was obtained from all the participants before initiation of the experiment.

### 2.2. Stimuli

Pre-recorded sentences from a Kannada sentence identification test were used [30]. The test comprises twenty-five standardized lists, each having ten sentences designed to be equivalent in difficulty, along with five practice lists. Each sentence in the list contains four key words. These target Kannada sentences were presented with a four-talker Kannada babble as the interfering noise. The target was spoken by a female talker, while the babble consisted of two female and two male talkers, and all stimuli were sampled at a rate of 44.1 kHz. The stimulus was further spatialized, and its ENV was enhanced as described below.

#### 2.2.1. Spatialization

The root-mean-square (RMS) amplitudes of the target speech and background noise were scaled to obtain the required SNRs of 5, 0 and −5 dB, as used in the current study. The target and noise signals were spatialized separately using a non-individualized Head Related Transfer Function (HRTF). The HRTF data includes recordings from various positions around the ear. To replicate CI processing, measurements from the behind the ear (BTE) front microphone configuration of the Oldenburg database were selected [31]. The non-individualized HRTF was used to ensure uniform spatial conditions across participants, with individual differences in pinna and head-related acoustics not explicitly considered.

**Figure 1:**
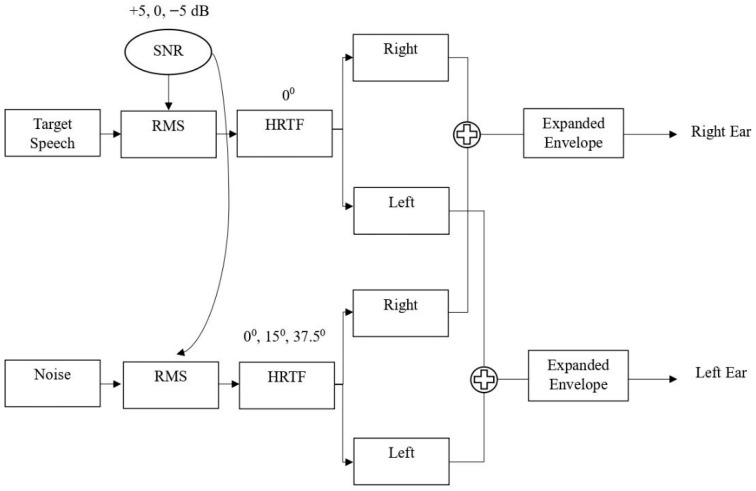
Block diagram representing the process of spatialization and ENV enhancement. RMS: root mean square; SNR: signal-to-noise ratio; HRTF: head-related transfer function.

Spatialization was performed by convolving the auditory signals with the appropriate HRTF impulse responses. For the target speech, the HRTF corresponding to 0° azimuth was used, and for the background noise, HRTFs at 0°, 15°, and 37.5° were employed. After HRTF processing, the spatialized noise and speech signals were combined by summing the respective right and left channel components, thereby preserving the binaural structure. The resulting binaural mixtures (speech + noise) were then processed using the ENV expansion algorithm. This enhancement was applied independently to each channel before being presented to the respective ears. Since ENV enhancement typically results in increased signal intensity, the analog output was adjusted so that the instantaneous amplitude will change but the overall ENV will remain the same after the enhancement. To match the overall RMS with and without enhancement, calibration was performed by comparing the sound pressure levels (SPL) of the signals before and after ENV enhancement using the NTi Audio XL 2 sound level meter. Figure 1 illustrates the block diagram representing the process of spatialization and ENV enhancement.

#### 2.2.2. Envelope Enhancement

The present study employed the compression-expansion method of ENV enhancement [18]. This approach was chosen based on evidence demonstrating its ability to improve the consonant-to-vowel ratio and reduce both forward and backward masking effects, thereby enhancing speech recognition in individuals with normal hearing as well as those with cochlear hearing loss.

The enhancement procedure was applied by expanding the amplitude of the speech + noise signal, *X*(*t*), using MATLAB R2023b (The MathWorks Inc., Natick, MA, USA) [32]. The signal *X*(*t*) was divided into four bands using 1024th order FIR band-pass filters. The cut-off frequencies were selected based on the Greenwood’s function. Full-wave rectification and low-pass filtering (3rd-order Butterworth filter) with a cut off frequency of 32 Hz was used to extract the temporal ENV, *E*(*t*), from each band. This cut-off frequency was selected based on the findings of a study by [7]. The ENV obtained was either left unchanged or modified by raising it to a power, denoted as *k*, which varied based on the instantaneous ENV amplitude *E*_i_. The value of *k* ranged from a maximum enhancement factor (*k*_max_ = 4) to a maximum compression factor (*k*_min_ = 0.3). This variation followed a decreasing exponential function relative to *E*_i_*,* such that: (i) the smallest ENV amplitude *E*_min_ received the highest enhancement (*k*_max_ = 4), and (ii). The largest ENV amplitude received the highest compression (*k*_min_ = 0.3).

The specific formula used to calculate *k* is presented below:

(1) $$k_{i}=e-\frac{\left(E_{i}-E_{min}\right)\;}{\tau }\;\left(k_{max}-k_{min}\right)+k_{min}$$

In this equation, τ remains a constant, set at 0.01 for each word in the present study. The minimum ENV amplitude *E*_min_ was calculated across the entire duration of the signal within the respective band. A correction factor was derived by taking the ratio of the enhanced ENV to the original ENV at each time point. This correction factor was then applied to the original band-pass filtered signal on a sample-by-sample basis. All the processed bands were subsequently summed to reconstruct the enhanced signal which was then passed through a third-order Butterworth low-pass filter with an 8000 Hz cut-off. Finally, the RMS amplitude of the enhanced signal was adjusted to match that of the original, unprocessed signal.

### 2.3. Procedure

The experiment was conducted in a sound-treated room with an ambient noise level of 20.8 dB(A). Participants were comfortably seated, and the auditory stimuli were delivered bilaterally using circumaural headphones (Sennheiser HD 280, Wedemark, Germany). The headphones were connected to personal laptop, with audio output routed through a Creative Sound Blaster X-Fi sound card. All signal processing, experimental setup, and stimulus presentation were carried out using MATLAB R2023b installed on the laptop.

Participants were instructed to carefully listen to each stimulus and repeat as many words from the sentence as they could recall. Responses were recorded and subsequently used for further analysis. Before the main experiment began, a familiarization session was provided using five practice lists, each containing 10 sentences. These practice sentences were presented at a SNR of +5 dB, with the target and noise spatially separated by 37.5°. Familiarization included both enhanced and non-enhanced versions of the stimuli, which were delivered at the participants’ most comfortable listening level.

Following familiarization, speech recognition performance was assessed under three spatial conditions: (1) a co-located condition where both the target and masker were presented at 0° azimuth, and (2) two spatially separated conditions in which the target remained at 0° while the masker was presented at 15° and 37.5°, respectively. Only positive azimuths (+15° and +37.5°) were tested because the stimuli were spatialized using non-individualized HRTFs in a simulated environment, resulting in ITD and ILD cues that are mathematically mirror-symmetrical across the left and right hemifields. This allowed greater experimental efficiency and reduced participant fatigue, while still providing a representative assessment of how the enhancement algorithm interacts with increasing spatial separation. To minimize order effects and learning bias, the order of conditions, SNRs, azimuths, and sentence lists was randomized for each participant. Sentence lists were not repeated across conditions. Each test list contained 40 keywords, with each correct keyword earning a score of 1, resulting in a maximum possible score of 40 per list. The entire testing session lasted approximately one and a half hours per participant

## 3. Results

The study examined how enhanced and non-enhanced temporal ENV conditions influenced spatial speech-in-noise perception at target-masker separations of 0°, 15°, and 37.5° azimuth across three SNRs (−5, 0, and +5 dB). Speech recognition scores obtained in each listening condition were used to calculate SRM values. Two SRM measures were derived: SRM15°, representing the difference in performance between 0° and 15°, and SRM37.5°, representing the difference between 0° and 37.5°. These measures quantified the extent to which spatial separation improved speech recognition across the different ENV conditions and SNR. The experiment was conducted at three SNRs: −5, 0, and +5 dB.

All statistical analyses were conducted in RStudio (version 2024.09.1) [33]. The modeling and diagnostic workflow employed the following packages: *glmmTMB* for fitting generalized linear mixed models (GLMMs) [34], *car* for Wald Chi-square tests [35], *DHARMa* for residual diagnostics and assumption checks [36], and *emmeans* for computing estimated marginal means (EMMs) and conducting pairwise comparisons [37]. Graphs were generated using *ggplot2* [38].

GLMMs were selected because they allow modeling of repeated-measures data while accounting for both fixed and random effects, as well as their interactions. This framework is particularly suited to speech-in-noise experiments, where multiple measurements per participant create non-independent observations. To examine the distribution of the outcome, probability density functions (PDFs) were plotted for combinations of condition, SNR, and SRM. The distributions were approximately normal, and a GLMM with a Gaussian distribution was fitted. Model assumptions were further evaluated using DHARMa diagnostic tests. The original SRM measures did not fully meet the assumptions of homoscedasticity and uniform residual distribution. To stabilize variance while retaining the polarity of values, SRM scores were subjected to a signed logarithmic transformation as given in the equation below.

(2) $${SRM}^{\prime }=sign(SRM)\;\cdot \;log(|SRM|+1)$$

Following the transformation, the dependent variable was analyzed using a Gaussian GLMM with identity link *SRM*′~ Condition* SNR*SRM_measure + (1|Subject ID). Subject ID was included as a random intercept to account for repeated measures within individuals. Levene’s test indicated that the variance of the transformed SRM values did not significantly differ across Condition (F = 2.55, *p* = 0.11), SNR (F = 0.81, *p* = 0.45), or SRM measure (F = 0.09, *p* = 0.76).

Further, DHARMa simulation-based residual diagnostics confirmed that model assumptions were met. The dispersion test showed no evidence of over- or under-dispersion (dispersion = 1.012, *p* = 0.859), and the Kolmogorov–Smirnov uniformity test suggested that residuals were approximately uniformly distributed (D = 0.071, *p* = 0.052). Together, these results indicated that the Gaussian GLMM provided an appropriate fit to the transformed data without requiring variance structure adjustments.

After verifying the model assumptions using DHARMa diagnostic tests, fixed, random, and interaction effects were then evaluated using Type II Wald Chi-square tests. The analysis revealed significant main effects of Condition (χ^2^(1) = 148.26, *p* < 0.001), SNR (χ^2^(2) = 535.95, *p* < 0.001), and SRM measure (χ^2^(1) = 12.10, *p* = 0.0005). The Condition × SNR (χ^2^(2) = 5.63, *p* = 0.06), Condition × SRM type (χ^2^(1) = 0.97, *p* = 0.33), SNR × SRM measure (χ^2^(2) = 1.90, *p* = 0.39), and the three-way interaction (χ^2^(2) = 2.91, *p* = 0.23) were not statistically significant.

**Figure 2:**
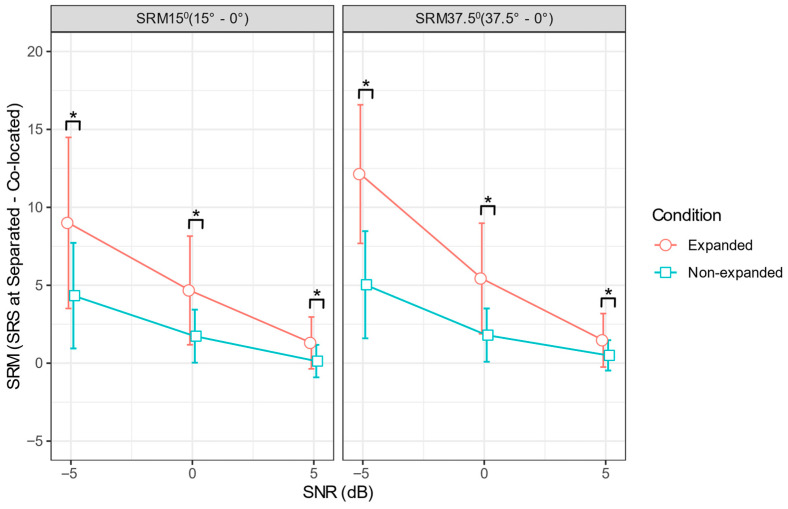
SRM at 15° (**left**) and 37.5° (**right**) target–masker separations as a function of SNR. Red circles represent the enhanced condition and blue squares the non–enhanced condition, with error bars showing ±1 standard error. Asterisks (*) denote statistically significant differences between the two conditions.

The primary objective of this study was to investigate the effect of ENV enhancement on SRM15° and SRM37.5°. Therefore, the pairwise comparisons were done separately for each SNR and SRM measure. For this purpose, the estimated marginal means from the fitted models were computed and subjected to further pairwise comparisons. The Bonferroni corrections were applied to account for the multiple comparisons. Across all SNR levels and both SRM measures, the enhanced condition consistently produced significantly higher SRM values than the non-enhanced condition (Figure 2). For SRM15°, the differences were statistically significant at all SNR levels, with *t*(346) = 4.583, *p* < 0.0001 at −5 dB SNR, *t*(346) = 4.945, *p* < 0.0001 at 0 dB SNR, and *t*(346) = 4.181, *p* < 0.0001 at 5 dB SNR. For SRM37.5°, the differences were likewise significant across all SNR levels, with *t*(346) = 6.565, *p* < 0.0001 at −5 dB SNR, *t*(346) = 6.524, *p* < 0.0001 at 0 dB SNR, and *t*(346) = 3.027, *p* = 0.0027 at 5 dB SNR. These results confirm that temporal ENV enhancement enhanced SRM under all tested conditions, with the greatest benefits observed at lower SNRs and wider spatial separations.

### 3.1. Objective Analysis of ITD and Interaural Coherence Using Cross-Correlation

To evaluate binaural temporal processing, objective analyses were conducted using the normalized cross-correlation function in MATLAB. The normalized cross-correlation *R_xy_*_,_*_coeff_*(*m*) was calculated using the following equation:

(3) $$R_{\mathrm{xy,coeff}}(m)\;=\;\frac{R_{xy}\left(m\right)}{\surd R_{xx}\left(0\right)R_{yy}(0)}$$

where *x* and *y* represent the right and left ear ENV signals respectively, and *m* = 1, 2, … 2*n* − 1, with *n* being greater of the lengths of *x* or *y*. *R_xy_*(*m*) denotes the cross-correlation between *x* and *y*, while *R_xx_*(0) and *R_yy_*(0) refer to the autocorrelations at zero lag. The absolute value of the peak of this function was defined as the IC, ranging from 0 (no similarity) to 1 (perfect similarity). The ITD was estimated from the lag position of this peak.

Stimuli included speech embedded in uncorrelated noise. The noise signals were independently generated for the left and right ears and verified as uncorrelated through paired t-tests and Pearson correlation, both of which indicated no significant similarity between the channels. Speech-shaped noise was scaled to produce SNRs ranging form −20 dB to +20 dB in 1 dB steps, resulting in 41 different SNR conditions. For each condition, the binaural signals were summed and analyzed to compute ITD and IC. The same procedure was repeated with ENV enhancement applied to the speech signal to assess the effect of modulation enhancement on binaural cue integrity.

### 3.2. Effect of SNR and ENV Enhancement on ITD and Interaural Coherence

The IC and ITD estimates varied systematically with changes in SNR. At higher SNRs, the cross-correlation function reflected strong binaural similarity, yielding high IC (values approaching 1) and stable ITD estimates that accurately reflected the underlying speech signal. In contrast, at negative SNRs, where the uncorrelated noise dominated the stimulus, IC values decline significantly, and ITD estimates became increasingly unreliable. These results indicate that the dominant uncorrelated noise in low SNR conditions disrupts temporal alignment across ears, making the cross-correlation measure driven more by random noise fluctuations than coherent speech structure.

**Figure 3:**
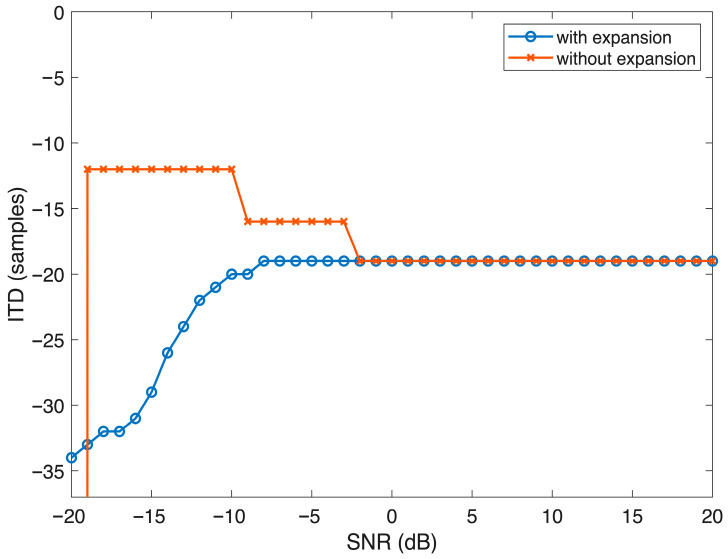
Graph depicting the estimation of ITD across different SNRs for the enhanced and non-enhanced signal.

**Figure 4:**
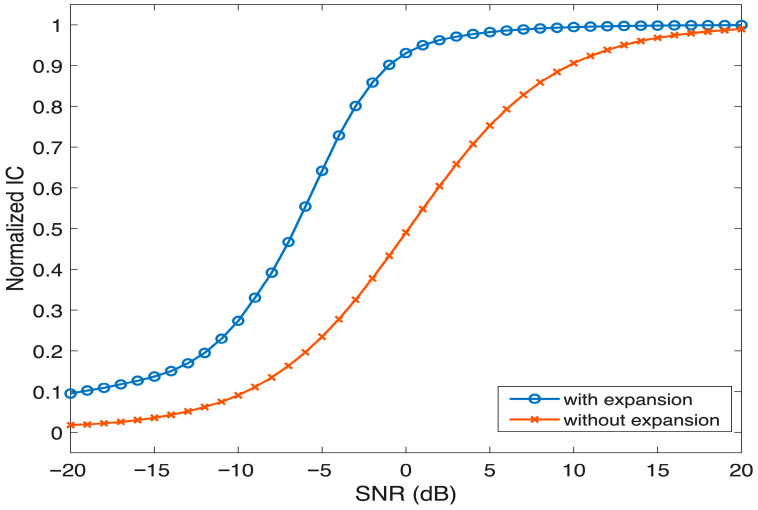
Graph depicting the IC across different SNRs for the enhanced and non-enhanced signal.

Application of the ENV enhancement scheme improved IC across a range of SNRs, particularly at moderate SNRs, where the speech signal was partially masked. Enhanced modulations appeared to increase the temporal salience of the speech ENV, aiding in the recovery of binaural cues. However, at extremely low SNRs, where the speech signal was masked by the noise, the benefits of ENV enhancement diminished, and ITD estimates remained inaccurate. The effect of ENV enhancement on ITD and IC is depicted in Figure 3 and Figure 4 respectively.

## 4. Discussion

The present study investigated the effects of ENV enhancement on SRM in normal hearing young adults. It also examined how this signal processing approach impacts acoustic correlates of binaural cues, specifically ITD estimates and IC across varying SNRs and azimuth separations. The findings show that ENV enhancement can enhance speech intelligibility, particularly under adverse listening conditions, and facilitate the use of spatial cues essential for segregating speech from background noise.

### 4.1. Effect of ENV Enhancement on Speech Recognition and SRM

Consistent with previous research, our results confirmed that spatial separation between target and masker and increasing SNR significantly improved speech recognition scores [39,40,41]. Notably, the interaction between ENV enhancement and spatial separation revealed that the benefits of spatial cues were more pronounced under the enhanced condition, especially at negative SNRs. This aligns with prior findings that SRM is typically greater under degraded listening conditions [2,42] where listeners rely more heavily on spatial cues to distinguish target speech from competing talkers.

In our study, at −5 dB SNR, SRM was significantly greater in the ENV-enhanced condition than in the non-enhanced condition at both 15° and 37.5° azimuths. This suggests that modulation enhancement plays a critical role in promoting spatial unmasking when the target signal is acoustically masked. However, at +5 dB SNR, the advantage diminished, likely reflecting a ceiling effect when ENV cues are already sufficiently prominent and further enhancement provides limited additional benefit. These results indicate that ENV enhancement is most beneficial under conditions with highest spatial processing demands. Such low SNRs (e.g., −5 dB) represent challenging yet realistic everyday listening situations, including social gatherings, classrooms, workplaces, and public spaces. Even normal-hearing listeners experience substantial difficulty under such conditions, while individuals with hearing impairment often function at effectively poorer SNRs due to reduced audibility, temporal processing deficits, and limited SRM. In individuals with moderate sensorineural hearing loss associated with outer hair cell dysfunction, reduced cochlear amplification, loss of compression, and degraded frequency selectivity further impair speech discrimination in noise. In this context, the present findings suggest that enhancing temporal ENV cues may be particularly beneficial under adverse listening conditions by reinforcing acoustic cues that support speech perception in noise.

This interpretation aligns with the theoretical rationale that ENV cues are essential for speech perception in noise [43,44], and suggests that enhancing these modulations can improve the acoustic salience of spatial cues such as ITD and ILD [45,46]. The clinical implications are particularly relevant for individuals with hearing loss, CI users, and older adults, who often show reduced SRM [47,48,49]. The reduced SRM observed in individuals with hearing impairment may be due to the fact that HAs can distort ITDs relative to those present in natural acoustic listening [50]. It has been reported that ITD and not just ILD are necessary to restore SRM in simulated CI listening and that they can regain access to SRM if appropriate ITD cues are restored [51]. Similar difficulties are noted in older adults, who frequently exhibit poorer ITD processing compared to younger adults [52,53]. Age related ITD processing deficits contribute to difficulties in sound localization and speech understanding in noisy environments, even for older adults with clinically normal hearing [54,55] leading to reduced spatial unmasking.

The findings of this study imply that ENV enhancement may offer a potential signal-processing approach to better preserve acoustic ITD cues, thereby supporting spatial unmasking. One key observation in this study is that, although SRM was significantly greater in the enhanced condition, the overall speech recognition scores were higher in the non-enhanced condition. This discrepancy may be due to participants not having sufficient exposure to or familiarity with the perceptual characteristics of the enhanced stimuli. It is likely that with additional training or adaptation time, listeners would become more accustomed to the enhanced signal and performance in the ENV enhanced condition could improve to match that of the non-enhanced condition.

### 4.2. Temporal ENV Enhancement and Binaural Cue Preservation

To further understand the mechanisms underlying the observed improvements in SRM, we examined ITD and IC using cross-correlation analysis. Significantly higher IC was observed in the enhanced condition, especially at moderate SNRs, indicating greater interaural similarity at the acoustic level. These findings are consistent with previous work showing that steeper and deeper modulations enhance ITD sensitivity [56].

Several studies support the idea that ENV shaping improves ITD processing. It has been demonstrated that increasing off-time duration, steepening ENV slopes, and raising peak level significantly enhanced ITD sensitivity in normal hearing listeners. Increasing modulation depth and ENV steepness has also been shown to reduce ITD thresholds, reinforcing the role of precise modulation cues [57]. Other work has observed that asymmetric onset shaping can alter perceived sound localization, although it does not affect the ITD just noticeable difference (JND), suggesting that ENV manipulation can impact spatial perception without necessarily improving sensitivity [58]. Robust ITD detection has been reported even with carrier frequency mismatches, highlighting the resilience of the auditory system to ENV distortions. [59]. In contrast, ENV-based ITD thresholds have been found to be significantly higher than those based on TFS, particularly in older adults, indicating limitations of ENV cues in this group [60]. Further, consonant-based ENV enhancement has been shown not to systematically affect ITD sensitivity, suggesting that not all forms of ENV manipulation are equally effective for spatial hearing [61].

It has been reported that, although ITD thresholds are generally poorer in CI users, they improve with enhanced ENV features such as increased off-time and slope [62]. Earlier studies demonstrated that ITD sensitivity in CI users improved most with transient stimuli like click trains, with ENV ITDs being more ambiguous than ILDs [63]. Increasing modulation depth and using dual-site stimulation across different cochlear locations significantly improved ITD detection, suggesting spatial integration across electrodes [64]. Furthermore, sharpening ENV peaks has been shown to halve ITD thresholds and enhance lateralization in CI simulations, even in reverberant environments [46]. Collectively, these findings support the potential of ENV-based processing strategies to spatial hearing by reinforcing temporal modulation cues. The results of the present study further add to these evidences by demonstrating that ENV enhancement improves enhance acoustic correlates of spatial unmasking and binaural timing measures in normal hearing listeners, especially under challenging listening conditions. It is important to acknowledge that these findings characterize the efficacy of the signal processing strategy in the acoustic domain. While this validates the algorithm’s ability to enhance spatial cues at the input stage, clinical validation with electrical stimulation is required to confirm how these benefits translate to the neural interface of a CI.

## 5. Conclusions

The present study provides evidence that temporal ENV enhancement can enhance SRM and support acoustic correlates of binaural processing, as reflected by improvements in cross-correlation-based ITD estimates and IC. These improvements in SRM were most evident at lower SNRs, where spatial cues are critical for speech segregation. The findings highlight the importance of ENV-based modulation shaping in reinforcing the temporal features of speech that are essential for spatial hearing at the acoustic level. By enhancing the clarity of these cues, ENV enhancement contributes to better speech understanding in noisy environments and enhances the auditory system’s ability to process spatial information. These results suggest that ENV enhancement may be a promising strategy for improving spatial hearing, with potential applications in auditory rehabilitation and in signal processing strategies of HAs and CIs. Further research should explore long term effects of training with ENV enhanced signals and evaluate their efficacy across clinical populations.

## Data Availability

The data presented in this study are available on request from the corresponding author.

## Supplementary Materials

The following supporting information can be downloaded at: https://www.mdpi.com/article/10.3390/audiolres16010026/s1, Table S1: Demographic characteristics and pure-tone average thresholds (PTA4) of the study participants. PTA4 represents the average hearing threshold at 0.5, 1, 2, and 4 kHz for each participant.

## Abbreviations

The following abbreviations are used in this manuscript:

ENV	Temporal Envelope
TFS	Temporal Fine Structure
CI	Cochlear Implant
HA	Hearing Aid
ITD	Inter-aural Time Difference
ILD	Inter-aural Level Difference
IC	Interaural coherence
SNR	Signal to Noise Ratio
SRM	Spatial Release from Masking
GLMM	Generalized Linear Mixed Model
DBM	Deep Band Modulation
HRTF	Head Related Transfer Function
RMS	Root Mean Square
JND	Just Noticeable Difference
BTE	Behind the Ear
SPL	Sound Pressure Level
SRS	Speech Recognition Scores
