# Supplementary material for: The Effect of Modulation Enhancement Scheme on Speech Recognition in Spatial Noise Among Young Adults with Normal Hearing
Source: Audiol Res. 2026 Feb 14;16(1):26. doi: 10.3390/audiolres16010026 (PMC12921754; doi:10.3390/audiolres16010026)
Supplement: Supplementary file 1 [file audiolres-16-00026-s001.zip › audiolres-3915517-supplementary.pdf]

Supplementary Material:

Table S1: Demographic characteristics and pure-tone average thresholds (PTA4) of the study participants. PTA4 represents the average hearing threshold at 0.5, 1, 2, and 4 kHz for each participant.

**Participant ID**	**Age (years)**	**Gender**	**PTA4 (dB HL)**
P01	28	Female	12.5
P02	26	Female	8.75
P03	24	Male	16.25
P04	22	Male	12.5
P05	28	Male	12.5
P06	24	Male	12.5
P07	34	Female	11.25
P08	21	Male	15
P09	28	Female	10
P10	23	Male	12.5
P11	23	Female	10
P12	26	Female	13.75
P13	29	Male	8.75
P14	32	Female	15
P15	19	Female	8.75
P16	22	Male	13.75
P17	24	Male	13.75
P18	31	Female	13.75
P19	31	Female	11.25
P20	31	Female	13.75
P21	32	Female	11.25
P22	28	Female	16.25
P23	23	Female	12.5
P24	34	Female	12.5
P25	20	Female	10
P26	24	Female	8.75
P27	22	Female	8.75
P28	20	Female	10
P29	25	Female	12.5
P30	22	Female	8.75
